# Sleep disorders and stress in children and adolescents with ASD

**DOI:** 10.1192/j.eurpsy.2025.2324

**Published:** 2025-08-26

**Authors:** A. Alvarez, N. Santamaria, V. Bote, R. Medina, J. A. Monreal, B. Sánchez, A. Hervás

**Affiliations:** 1 Mental Health, University Hospital Mutua Terrassa, Terrassa, Spain

## Abstract

**Introduction:**

Sleep disorders are very common in children with neurodevelopmental disorders such as autism. Poor sleep can have detrimental effects on cognitive processes, attention, memory, language, and regulation of mood and behavior. Many people with autism have difficulty correctly processing sensory information that reaches them from both the environment and their own body. Depending on how the person with autism perceives the sensations, these can impact on sleep, causing nighttime awakenings or poor sleep conciliation. It is vitally important to evaluate nighttime rest and have knowledge of appropriate tools to improve sleep in patients with ASD.

**Objectives:**

In this work we aim to evaluate, in a child-youth population diagnosed with ASD who have been admitted to our ASD Day Hospital during the year 2024, the prevalence of sleep disorders and their characteristics. As well as generating strategies to improve sleep conciliation and factors to take into account or avoid that may be affecting our patients.

**Methods:**

A review is carried out of the clinical history of patients admitted to the TEA Day Hospital during the year 2024. Sleep problems are analyzed, as well as their relationship with stress. Results and measures to improve the quality of night rest in these patients are presented.

**Results:**

From January to September 2024, a total of 26 patients with autism have been treated at the ASD Day Hospital of the University Hospital of Mutua Terrasa, in the following programs: intensive, eating problems and low functionality. Of these, 32% were women, with an average age of 11.64 and an average stay of 40 days.

More than half of the patients had sleep problems, where the majority had more than one. Among the most frequent: difficulties falling asleep (12), maintenance insomnia (8), frequent awakenings (4), night terrors (6), and the need for the presence of parents and/or sensory interventions to be able to fall asleep (12).

The main interventions carried out have consisted of: establishing adequate sleeping habits, avoiding naps, avoiding highly stimulating activities, feeling tired beforehand, adapting environmental conditions to the needs of the child and giving proprioceptive information before sleeping.

Among the most commonly used drugs we found atypical antipsychotics: olanzapine, risperidone. Some antidepressants: mirtazapine and occasionally benzodiazepines. The vast majority of children took melatonin.

**Conclusions:**

Sleep disorders are highly prevalent in patients with ASD, but correct intervention can improve comorbid symptoms such as anxiety, stress and discomfort, which are largely related to poor sleep.

Sensory-perceptive interventions are essential to improve nighttime rest in children with ASD

**Disclosure of Interest:**

None Declared

